# A PDMS-Al Triboelectric Nanogenerator Using Two-Pulse Laser to Enhance Effective Contact Area and Its Application

**DOI:** 10.3390/polym16233397

**Published:** 2024-12-02

**Authors:** You-Jun Huang, Chi-Hung Tsai, Chia-Wei Hung, Chen-Kuei Chung

**Affiliations:** Department of Mechanical Engineering, National Cheng Kung University, Tainan 701, Taiwan

**Keywords:** mechanical energy harvesters, triboelectric nanogenerators, polydimethylsiloxane, human–machine interface, polymer

## Abstract

A triboelectric nanogenerator (TENG) is a kind of energy harvester which converts mechanical energy into electrical energy with electron transfer and transport between two different materials during cycling tribology. To increase the contact area between tribo-layers and enhance the output of TENGs, many studies prepare patterned micro/nanostructured tribo-layers using semiconductor processes like lithography and etching at high cost and with long processing times. Here, we propose a new method to quickly produce high-aspect-ratio (HAR) microneedles of polydimethylsiloxane (PDMS) for TENG triboelectric layers using a two-pulse laser-ablated polymethyl methacrylate mold and casting. It has the merit of employing low-cost CO_2_ laser microfabrication and polymer casting in a feasible way to produce efficient tribo-electric layers. Two-pulse laser ablation is an efficient method for fabricating HAR microstructures with increasing depth at a constant width and density compared to single-pulse ablation. It enhances the depth of microneedles at a constant width and successfully casts PDMS tribo-layers with microneedles that have an aspect ratio 1.88 times higher than those produced by the traditional single-pulse process. The microneedle-PDMS (MN-PDMS) layer is combined with Al sheets to form the MN-PDMS-Al TENG. Compared with the flat PDMS-Al TENG and single-pulse PDMS-Al TENG, the two-pulse TENG enhances open-circuit voltage (V_oc_) by 1.63 and 1.48 times, the short-circuit current (I_sc_) by 1.92 and 1.47 times, and the output power by 3.69 and 2.16 times, respectively. This two-pulse ablation method promotes the output performance of TENGs, which has the potential for applications in self-powered devices and sustainable energy.

## 1. Introduction

A triboelectric nanogenerator (TENG) is a kind of energy harvester which converts mechanical energy into electrical energy with electron transfer and transport between two different materials during cycling tribology [1,2,3,4]. Traditionally, there are two approaches to enhancing the performance of TENGs: altering the material properties [5,6,7,8] and modifying the effective contact area [8,9,10,11,12]. Compared to material modifications, altering the surface microstructure of TENGs can also improve their mechanical sensitivity. Conventionally, scientists have employed semiconductor processes (e.g., photolithography and etching) to increase the effective contact area of TENGs [13,14,15,16]. In contrast, laser processing and molding offer advantages such as low cost, short processing time, environmental friendliness, and reusable molds [17,18,19]. The morphology of the TENG microstructures fabricated via laser processing and molding depends on the energy received by each spot during laser processing [17,20,21]. As the energy increases, the depth of the spot also increases, resulting in greater height and effective contact area of the TENG microstructure. However, with increasing energy [22,23], the base area of each spot also enlarges, limiting the density of laser-processed TENG microstructures to the depth of the microstructure and thereby restricting the design freedom of the height and effective surface area of laser-processed TENGs. In studies on laser processing microchannels, scientists have restricted the increase in the base area of spots through multiple processing cycles of the same spot, thus preparing laser-processed microstructures with higher aspect ratios and offering more possibilities for laser-processed microstructures.

In this study, a two-step laser processing TENG with a high aspect ratio was designed. Through two-pulse laser ablation of a polymethyl methacrylate (PMMA) mold, laser microstructures with higher aspect ratios compared to single-pulse laser ablation were etched on the PMMA mold. Subsequently, PDMS tribo-layers with micro-needle structures (TPL-PDMS) were prepared through molding, which, combined with low-purity aluminum, formed friction pairs to constitute the two-pulse laser-enhanced PDMS-Al TENG (TPL-PDMS TENG). Compared to traditional single-pulse laser-processed TENGs (SPL-PDMS TENGs), TPL-PDMS TENGs exhibited an aspect ratio that was 1.88 times higher. Compared to the flat PDMS-Al TENG and SPL-PDMS TENG, the TPL-PDMS TENG showed 1.63 times and 1.48 times higher open-circuit voltage, 1.92 times and 1.47 times higher short-circuit current, and 3.69 times and 2.16 times higher output power, respectively. Laser processing combined with polymer molding is a low-cost, rapid, and repeatable technique. Additionally, the TPL-PDMS TENG holds potential for driving electronic devices and providing clean and sustainable energy. Additionally, the TPL-PDMS TENG can drive 500 LEDs, while the SPL-PDMS TENG drives 300 LEDs, and the flat PDMS-Al TENG drives 270 LEDs. When applied as a self-powered human motion sensor, the TPL-PDMS TENG captured voltage signals of 1.34 V from wrist movement and 4.56 V from elbow movement. By integrating this with a circuit system and microcontroller, a rehabilitation assistance counter was created to record the number of correct rehabilitation movements and provide alerts when movements are incorrect or when set targets are reached. This system can significantly reduce the labor costs associated with basic rehabilitation if widely implemented.

## 2. Experimental Procedures

### 2.1. Design and Fabrication of TPL-PDMS-TENG and SPL-PDMS-TENG

To measure the differences in electrical output performance and morphology of PDMS microstructures produced by single-pulse and two-pulse laser processing, two different depths of PMMA master molds were designed to prepare two types of PDMS tribo-layers. Firstly, a CO_2_ laser (LST Group VL2000 advanced, Punchbowl, Australia) suitable for engraving PMMA was used to engrave 100 rows of microstructures on a 50*50*5 mm^3^ PMMA substrate at a power of 1.5 W, a cutting speed of 39.9 mm/s, and 50 pulses per inch. Another PMMA of the same size was processed twice using the same parameters. Both PMMA substrates were surrounded by heat-resistant tape to achieve a targeted tribo-layer thickness of 2 mm. Subsequently, two portions of 11 g PDMS were mixed at a 10:1 ratio of base to curing agent and poured into the PMMA molds. The molds were then placed in a vacuum chamber at 70 mTorr for 10 min to remove bubbles between the PMMA mold and the PDMS. The PMMA molds with PDMS were then heated and cured in an 80 °C oven for 1 h. After cooling, the molds were demolded to obtain the SPL-PDMS tribo-layer and TPL-PDMS tribo-layer, respectively. These tribo-layers were individually attached to low-purity aluminum to form tribo-pairs, resulting in an SPL-PDMS-TENG and a TPL-PDMS-TENG, respectively. Additionally, a piece of PDMS with the same dimensions but without structures was assembled into a flat PDMS TENG as a control.

### 2.2. The Material Characterization and Electrical Measurements

To observe and compare the microstructural morphology of SPL-PDMS tribo-layers and TPL-PDMS tribo-layers, optical microscopy (OM, Olympus BX 51 M, Tokyo, Japan) images were used. The output electrical performance of TENGs is primarily characterized by open-circuit voltage (Voc) and short-circuit current (Isc). To compare the output electrical performance of different TENGs, an oscilloscope (HIOKI Memory HiCorder MR8870-20, Nagano, Japan) was used to capture the electrical outputs, while a pneumatic cylinder actuation platform provided stable and continuous external force. On the platform, the PDMS tribo-layer of the TENG was fixed to the actuator shaft of the pneumatic cylinder (FESTO D: S-PAZ-DW20-100 PPV, Esslingen, Germany) via an acrylic plate, with the aluminum layer placed on a stationary plastic plate. Measurements were conducted with a stroke of 25 mm under conditions of 16 N and 7 Hz.

## 3. Results and Discussions

**Figure 1:**
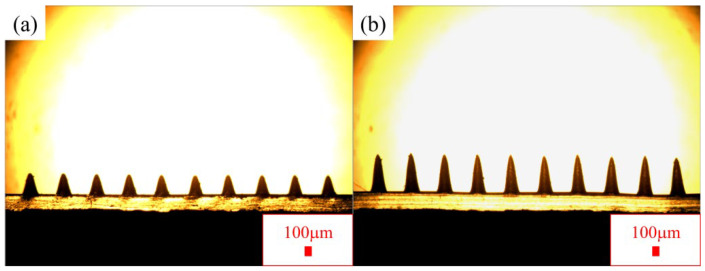
Two types of PDMS microneedle structures processed via laser were captured through OM imaging: (**a**) The SPL-PDMS tribo-layer, produced with a single laser pass, shows a height of 320 μm and a surface area of 3369 mm^2^ at a width of 220 μm; (**b**) the TPL-PDMS tribo-layer, produced with a double laser pass, achieves a height of 600 μm (1.88 times greater) and a surface area of 4308 mm^2^ (1.28 times greater) at the same width.

To observe the morphological differences between SPL-PDMS tribo-layers and TPL-PDMS tribo-layers, a row of microstructures was cut from each of the molded SPL-PDMS and TPL-PDMS tribo-layers and placed on slides for examination. Observations under an OM revealed the structural morphologies shown in Figure 1a,b. The measured average widths and heights of the structures were as follows: for the SPL-PDMS tribo-layer, width of 220 μm, height of 320 μm; for TPL-PDMS tribo-layer, width of 220 μm, height of 600 μm. From the above results, it can be observed that as the processing times increase, the structure’s width remains the same for single-pulse laser and two-pulse laser processing, while the height increases from 320 μm to 600 μm, a 1.88-fold increase. The main reason for this is that during multiple processing cycles, in the second processing cycle, the laser beam falls on the inclined wall rather than the flat surface from the first processing cycle. The inclination of the holes increases after each processing cycle, resulting in a reduction in the taper of the channels. As the hole inclination increases, the amount of light reflected from the hole’s sidewall increases, and the surface absorption rate decreases. This leads to a reduction in the amount of material evaporating from the sidewall, keeping the channel width constant while the depth slope continues to increase.

Furthermore, to integrate the application of a friction generator, the calculated surface area of the friction layer based on the measured structural dimensions is presented in Table 1. It can be observed that the TPL-PDMS tribo-layer has 1.28 times the contact area compared to the SPL-PDMS tribo-layer. Combining the experimental results mentioned above, this study suggests that by using different processing frequencies with a fixed processing power, it is possible to fabricate microneedle structures with high aspect ratios and larger surface areas. The slender microneedle structures exhibit greater flexibility during deformation. This not only enhances the effective contact area and deformation capability of the friction generator but also extends the lifespan of the laser processing equipment. This approach contributes to improving the economic efficiency and meeting environmental sustainability requirements.

**Figure 2:**
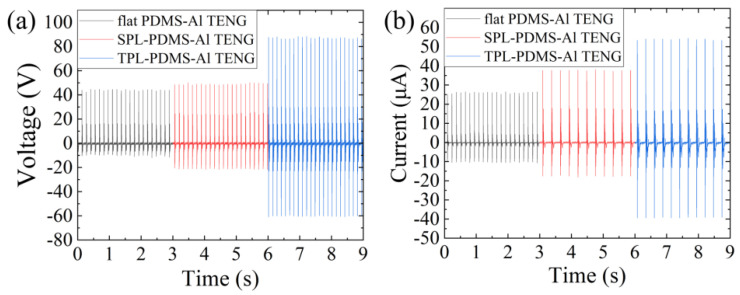
Output signal differences among the three TENG types: (**a**) the Voc for the flat PDMS-Al TENG, SPL PDMS-Al TENG, and TPL PDMS-Al TENG are 54.2 V, 59.7 V, and 88.4 V, respectively; (**b**) the Isc for the flat PDMS-Al TENG, SPL PDMS-Al TENG, and TPL PDMS-Al TENG are 26.5 μA, 34.5 μA, and 50.9 μA, respectively. It can be observed that the TPL PDMS-Al TENG shows a significant enhancement in both Voc and Isc compared to the flat PDMS-Al TENG and SPL PDMS-Al TENG.

Using the combination of a pneumatic cylinder actuation platform and an oscilloscope, we measured the Voc and Isc of different TENGs as references for evaluating their output performance, as shown in Figure 2a,b. The flat PDMS-Al TENG exhibited a maximum open-circuit voltage of 54.2 V, with a maximum output current of 26.5 μA under a 1 MΩ load. The SPL-PDMS TENG exhibited a maximum open-circuit voltage of 59.7 V, with a maximum output current of 34.5 μA under a 1 MΩ load. The TPL-PDMS TENG exhibited a maximum open-circuit voltage of 88.4 V, with a maximum output current of 50.9 μA under a 1 MΩ load. According to Table 2, it can be observed that the TPL-PDMS TENG has 1.63 times and 1.48 times the voltage output of flat PDMS-Al TENG and SPL-PDMS TENG, respectively. In terms of current output, it is 1.92 times and 1.47 times that of the flat PDMS-Al TENG and the SPL-PDMS TENG, respectively. In summary, increasing the processing times increases the surface area of the friction layer. Additionally, the finer and more flexible structure makes the effective contact area of the TENG higher, effectively enhancing the output performance of the TENG.

**Figure 3:**
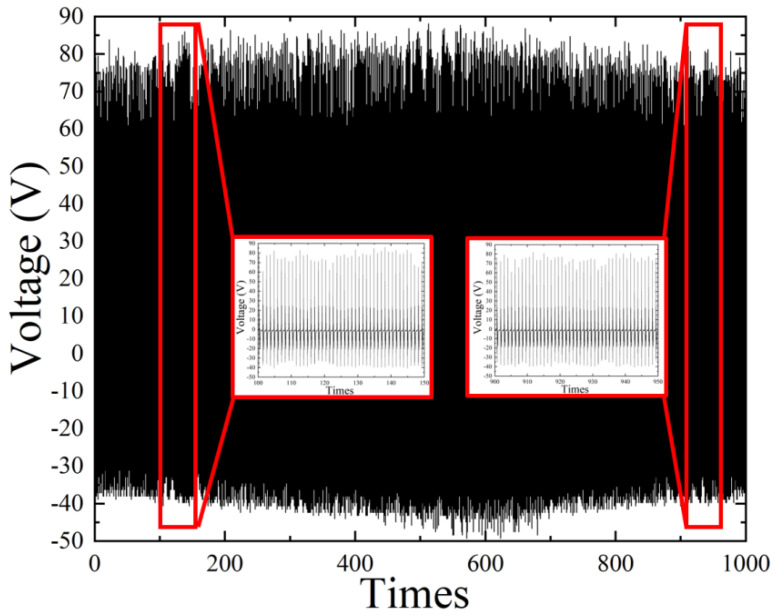
Output waveform of TPL-PDMS TENG during continuous operation on a pneumatic cylinder actuation platform for 1000 times, demonstrating that the output voltage remains stable without significant variation.

To ensure the durability of the TPL-PDMS TENG, we subjected it to 1000 cycles of operation on a pneumatic cylinder actuation platform. The results confirm that the TPL-PDMS TENG maintained stable output performance after 1000 cycles, as shown in Figure 3.

**Figure 4:**
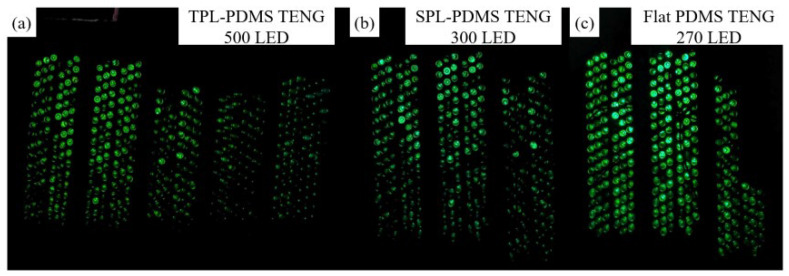
Number of LEDs driven by different PDMS TENGs: (**a**) TPL-PDMS TENG driving 500 LEDs, (**b**) SPL-PDMS TENG driving 300 LEDs, (**c**) flat PDMS TENG driving 270 LEDs.

In order to investigate the performance of the TENG under external loads, the voltage and current trends of TPL-PDMS TNEG were measured under different load resistances (10 KΩ, 100 KΩ, 1 MΩ, 5 MΩ, 10 MΩ), and the power output was calculated. The voltage increases with the increase in load resistance, while the output current decreases due to Ohmic losses. Subsequently, the power output was calculated using Ohm’s law (Equation (1)):

P = I^2^ × R (1)

where P is the instantaneous electrical power of the friction generator, I is the maximum measured current through the external resistance, and R is the load resistance value. As the results show, the maximum output power of TPL-PDMS TENG reaches 8.28 mW under a 1 MΩ load resistance. The TPL-PDMS TENG, SPL-PDMS TENG, and flat PDMS TENG were able to light up 500, 300, and 270 LEDs, respectively, as shown in Figure 4. This demonstrates the significant enhancement in the electronic driving performance of PDMS TENG achieved through the two-pulse laser process.

**Figure 5:**
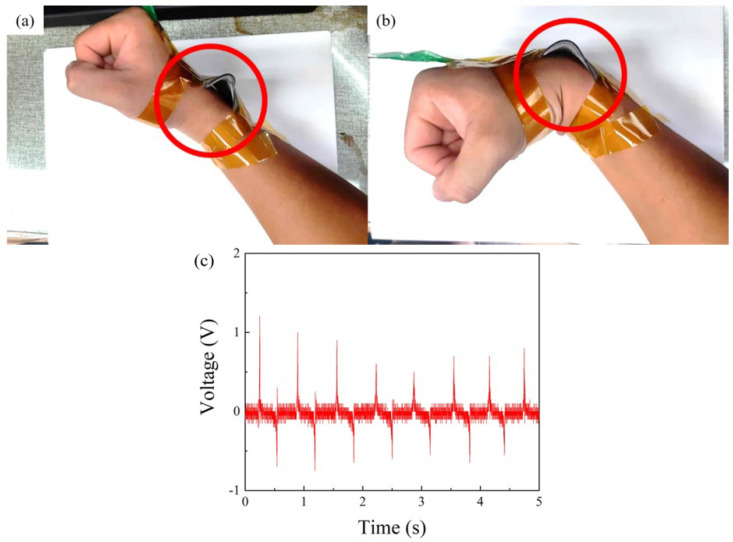
When the TPL-PDMS tribo-layer is used as a biomechanical sensor, it captures wrist flexion and extension signals. (**a**) When the subject’s wrist is extended, the TPL-PDMS tribo-layer separates from the skin, as shown in the red-circled area; (**b**) when the wrist flexes, the TPL-PDMS tribo-layer contacts the skin, as shown in the red-circled area, completing the contact–separation cycle; (**c**) voltage output graph of wrist flexion and extension detected by the TPL-PDMS tribo-layer, generating a peak signal of up to 1.34 V.

**Figure 6:**
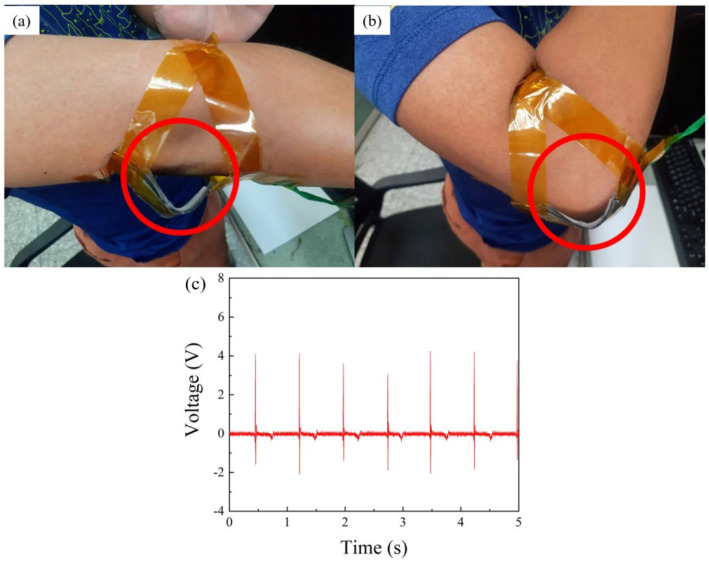
When the TPL-PDMS tribo-layer is used as a biomechanical sensor, it captures elbow flexion and extension signals. (**a**) When the subject’s elbow is extended, the TPL-PDMS tribo-layer separates from the skin, as shown in the red-circled area; (**b**) when the elbow is flexed, the TPL-PDMS tribo-layer contacts the skin, as shown in the red-circled area, completing the contact–separation cycle; (**c**) voltage output graph of elbow flexion and extension detected by the TPL-PDMS tribo-layer, generating a peak signal of up to 4.56 V.

Based on previous experimental results, it was determined that TPL-PDMS TENGs exhibit high output performance, stability, and sensitivity. We aim to apply this TENG to detect human hand joint movements. Since skin is positively charged in the triboelectric series, we chose skin as the positive electrode material. The TPL-PDMS tribo-layer created in this study acts as a single-electrode triboelectric layer, generating electrical transfer when in contact with the skin during human joint movements. This motion leads to the separation of the structures from the skin, resulting in the generation of voltage signals which serve as the basis for determining joint movements. Additionally, the output signals from the elbow and wrist are used for monitoring rehabilitation in cases of stroke and hand fractures. TPL-PDMS tribo-layers were placed separately on the wrist, forearm, and palm for motion detection. The triboelectric layer used in this study measures 508 mm × 508 mm, and aluminum foil was employed as the electrode for the single-electrode triboelectric layer fixed on the wrist and forearm. In the palm region, a complete TPL-PDMS TENG, similar to the vertical contact–separation TENG used earlier, was utilized. Subsequently, output signals were tested at different locations. Firstly, as a single-electrode triboelectric layer, the TPL-PDMS tribo-layer was placed on the wrist and subjected to bending and stretching, as illustrated in Figure 5. As seen in Figure 5a,b, with wrist movement, the TPL-PDMS tribo-layer contacted the skin, underwent triboelectrification, and separated while extending, forming a complete power generation cycle and generating a voltage waveform. The maximum output voltage during wrist movement was 1.34 V, as shown in Figure 5c. Next, the TPL-PDMS tribo-layer, as a single-electrode triboelectric layer, was placed on the elbow and subjected to bending and stretching, as shown in Figure 6. As seen in Figure 6a,b, with elbow flexing, the processed PDMS microneedle structure contacted the skin, and then separated with elbow extending, forming a complete power generation cycle and generating a voltage waveform. The maximum output voltage at the elbow was 4.56 V, as shown in Figure 6c. The main reason for this is the greater pressure applied to the TPL-PDMS tribo-layer during elbow bending. The signals collected during the movement of the TPL-PDMS tribo-layer were integrated through a circuit, combining the TENG with an Arduino UNO control board as an MCU for pressure sensing tests. Simultaneously, the pressure voltage sensing instrument was developed into an intelligent sensor and applied in human rehabilitation. When patients use the TPL-PDMS tribo-layer for cyclic movements, the system begins collecting signals. The collected signals are transmitted to the Arduino UNO for counting. When the specified count is reached, the buzzer and LED indicator will remind the rehabilitation patient that the target count has been achieved. In the Arduino UNO, we can set parameters to capture signals to avoid inaccurate rehabilitation movements. This setup allows for the collection of valid values to assist in rehabilitation. Due to the high sensitivity of the TPL-PDMS tribo-layer, the output signal values vary based on different pressures during rehabilitation. This characteristic helps patients monitor rehabilitation counts and related information about the recovery of the affected area.

In the aforementioned tests, we developed a single-electrode TENG capable of sensing human motion signals and applied it to assist in the rehabilitation of stroke or fracture patients. The system collects relevant signals during cyclic rehabilitation movements, allowing patients to monitor their rehabilitation counts and recovery status of the affected area while also reducing the waste of medical resources.

## 4. Conclusions

In this study, we developed a TPL-PDMS TENG with high-aspect-ratio microneedle structures using a dual-laser process, achieving this at a low cost and within a short time frame. The TPL-PDMS TENG exhibited a Voc of 88.4 V, an Isc of 50.9 μA, and an output power of 8.28 mW, outperforming the traditional single-laser fabricated SPL-PDMS TENG and flat PDMS TENG with 1.63 and 1.48 times the Voc, 1.92 and 1.48 times the Isc, and 3.69 and 2.19 times the power output, respectively. The aspect ratio and effective contact area of the TPL-PDMS TENG were 1.88 and 1.28 times greater than those of the SPL-PDMS TENG. Additionally, the TPL-PDMS TENG could power 500 LEDs, compared to 300 LEDs for the SPL-PDMS TENG and 270 LEDs for the flat PDMS TENG. When applied to human motion sensing, the TPL-PDMS TENG captured signals of 1.34 V from wrist bending and 4.56 V from elbow bending. This system was integrated with an Arduino UNO, buzzer, and LED in a rehabilitation assistance device that helps users monitor the accuracy and frequency of their rehabilitation movements, providing alerts or warnings for incorrect actions, and potentially reducing labor costs in rehabilitation.

## Figures and Tables

**Table 1:** PDMS microneedle structure dimensions and contact surface area.

Figure	Figure 1a	Figure 1b
Number of pulses	1.5 W × 1	1.5 W × 2
Width (μm)	220	220
Height (μm)	320	600
Surface area (mm^2^)	3369	4308

**Table 2:** Comparison of the outputs of three different types of TENG in this study.

Power (W)	Number of Pulses	Speed (mm/s)	PPI	Surface Area (mm^2^)	V_oc_ (V)	I_sc_ (μA)
flat					54.2	26.5
1.5	1	39.9	50	3369	59.7	34.5
	2			4308	88.4	50.9

## Data Availability

Data are presented in the coauthors’ research results and schematic drawings available on request.
